# Editorial: Multi-omics strategies to analyze complex agronomic traits in plants, volume II

**DOI:** 10.3389/fpls.2025.1614041

**Published:** 2025-05-09

**Authors:** Guo-Fei Tan, Lin Chen

**Affiliations:** ^1^ Institute of Horticulture, Guizhou Academy of Agricultural Sciences (GAAS), Ministry of Agriculture and Rural Affairs Key Lab of Crop Genetic Resources and Germplasm Innovation in Karst Region, Guiyang, Guizhou, China; ^2^ Institute of Animal Science, Chinese Academy of Agricultural, Sciences, Beijing, China

**Keywords:** multi-omics, genetic selection, agronomic traits, crop improvement, breeding

Multi-omics (such as genomics, transcriptome, metabolomics, proteomics, and others) analysis is a strategy integrating multiple omics techniques for research. It is an essential and effective method for studying complex biological traits, which can provide a more comprehensive and in-depth understanding of the functions and mechanisms of biological systems and provide more accurate information for the study of complex agricultural traits in plants. On the basis of the previous volume, we continue to explore the analysis of complex agronomic traits by multi-omics, hoping to apply the latest multi-omics techniques to the analysis of complex agronomic traits and provide a reference for plants.

In volume II, a total of 6 original research articles and 1 review article were selected from 15 manuscripts focused on this topic. These six research articles reported research on *Zea mays* (maize, two articles), *Citrullus lanatus* (watermelon, two articles), *Rubus idaeus* (red raspberry, one article), and *Stipa breviflora* (one article); the review article concerns the transcriptional regulatory landscape of crops.

Among these articles, research focused on the agronomic traits related to plant development, regulation, and postharvest. The multi-omics strategies included metabolome, transcriptome, and GWAS and bisulfide-assisted sequencing (BAS-seq) methods. For the benefit of potential readers, the key points of the seven articles in this Research Topic are highlighted as follows:

Maize is a global crop for which the photoperiod sensitivity and heterosis are important to environmental adaptation and yield. Jiang et al. have identified genetic loci and candidate genes for photoperiod sensitivity by integrating GWAS, linkage, and transcriptome analysis. The results showed that a total of 48 quantitative trait loci (QTLs) and 252 quantitative trait nucleotides (QTNs) were detected by linkage population and the inbred association panel, and 13 candidate genes were identified by GWAS, linkage analysis, and transcriptome analysis. *MYB163*, *bif1*, *burp8*, *CADR3*, and *Zm00001d050238* were the candidate genes for photoperiod sensitivity. For maize heterosis under drought stress, Dai et al. used Zhengdan7137 and Zhengdan7153—two maize hybrid varieties—as materials under well-watering (WW), water-deficit (WD), and re-watering (RW) conditions for RNA-Seq analysis. The results showed a total of 303 and 252 conservative drought response overdominance genes (DODGs) and underdominance genes (DUDGs) were identified, respectively, and 165 and 267 conservative re-watering response overdominance genes (RODGs) and underdominance genes (RUDGs), respectively. These results provide important candidate genes for further molecular mechanism research of photoperiod sensitivity and heterosis under drought stress in maize.

Stripe margin pattern and rind color of watermelon peel are the significant traits that affect consumer acceptability. In the peel stripe margin pattern of watermelon, Yang et al. identified 44 transcription factor families and candidate genes believed to regulate the stripe margin trait of watermelon peel. The *ClMYB36* and *ClAPRR5* genes might be involved in regulating the peel stripe margin and modulating the blurred peel stripe margin, respectively, by fine genetic mapping and transcriptomic analysis. In the rind color of watermelon, Liu et al. revealed that a single major gene, *Cla97C08G161570*, is the dominant single gene that regulates the major genetic locus associated with the dark green rind trait by genetic segregation, whole-genome BAS-seq analysis, and pairwise sequence comparisons analysis. A total of 940 differentially expressed genes (DEGs) associated with rind coloration were identified by comparative transcriptomes. *C01G023430*, *C04G071470*, *C09G165830*, *C07G128820*, *C08G148460*, and *C08G155040* were identified as the key genes, and they exhibited high levels of differential expression as seen using GO and KEGG enrichment analysis. These results provide important candidate genes for further molecular mechanism research of watermelon peel color.

Anthocyanins are the main component of red raspberry fruit, and the mechanism of anthocyanin synthesis in postharvest at various color stages is unclear. Sun et al. identified 43 key metabolites and 13,239 DEGs linked to anthocyanin biosynthesis in postharvest raspberry color development based on targeted metabolomic analysis. A total of 9 structural genes, 19 transcription factor genes, and 14 hormone signaling genes involved in the biosynthesis of cyanidin-3-*O*-sophoroside were also identified by transcriptome and metabolome. The research provides insights into the synthesis of anthocyanins during the coloration of postharvest raspberry fruit.

*Stipa breviflora* is a dominant species in the desert steppe of Northern China, and grazing intensities can cause morphological characteristics and transcriptional regulation to differ in *S. breviflora* plants. Wang et al. revealed that epidermis cells and xylems significantly thicken with grazing intensity, and the components of cell walls were all increased dramatically and significantly under both moderate and heavy grazing by anatomical analysis. Transcriptome analysis showed that the differentially expressed genes related to different grazing intensities were also engaged in plant cell wall formation, photosynthesis, and respiration. This research points out that appropriate grazing density is of great significance for maintaining the normal life growth and development of *S. breviflora.*

Crop breeding entails developing and selecting plant varieties with improved agronomic traits, and the integration of multi-omics data has been proven to be crucial for the construction of high-confidence regulatory networks. Huo et al. summarized how these omics technologies can be applied to specific agronomic research and breeding improvement, and they focus on the transcriptional regulatory/gene regulatory network landscape of crops. This review provides a better understanding of multi-omics in improving crop breeding efforts and contributing to global food security.

Based on the articles published in this Research Topic, research tactics of complex crop agronomic traits with multi-omics techniques have been introduced, which not only requires joint analysis of multiple omics but also requires analysis of population genetics, morphology, physiology, cells, and other data to better conduct comprehensive, accurate, and rapid analysis of complex agronomic traits in plants. With the continuous changes in environmental climate and human needs, the requirements for special agricultural traits of crops in the future are becoming increasingly diverse. We hope that a comprehensive analysis of multi-omics can help readers and researchers to study the development needs of omics in the future. We are very grateful for the efforts made by journal editors, peer reviewers, and relevant authors. Without their help, this Research Topic would not be presented to the readers. We would also like to thank all colleagues who contributed to this Research Topic. We hope that readers can obtain valuable information from this topic to help them achieve success in the future.

